# Improvement of functioning in patients with schizophrenia: real-world effectiveness of aripiprazole once-monthly (REACT study)

**DOI:** 10.1186/s12888-023-04893-8

**Published:** 2023-05-31

**Authors:** Oloruntoba Oluboka, Guerline Clerzius, Wolfgang Janetzky, Daniel Schöttle, François Therrien, Klaus Wiedemann, Marc-André Roy

**Affiliations:** 1 University of Calgary, 2500 University Drive NW, Calgary, AB T2N 1N4 Canada; 2 Lundbeck Canada Inc, 2600 Alfred-Nobel Boulevard, Saint-Laurent, QC H4S 0A9 Canada; 3 Lundbeck GmbH, Ericusspitze 2, 20457 Hamburg, Germany; 4 Klinik für Psychiatrie und Psychotherapie, Zentrum für Psychosoziale Medizin, Universitätsklinikum Hamburg-Eppendorf, Martinistrasse 52, 20246 Hamburg, Germany; 5 Otsuka Canada Pharmaceutical Inc., 2250 Alfred-Nobel Boulevard, Saint-Laurent, QC H4S 2C9 Canada; 6 Département de Psychiatrie et Neurosciences, Faculté de Médecine, Centre de recherche CERVO, Université Laval, Clinique Notre-Dame des Victoires, 2525, chemin de la Canardière Porte, A-1-2, Québec, G1J 2G3 Canada

**Keywords:** Schizophrenia, Long-acting injectable, Observational study, Real-world evidence, Aripiprazole once-monthly, Functioning

## Abstract

**Background:**

Functional impairment affects many patients with schizophrenia. Treatment with the long-acting injectable antipsychotic aripiprazole once-monthly (AOM) may help improve functioning.

**Objectives:**

To explore changes in functioning in patients with schizophrenia who received AOM treatment in observational studies.

**Methods:**

Here we report functional outcomes in the form of Global Assessment of Functioning (GAF) scores in a pooled analysis of data from two observational studies from Canada (NCT02131415) and Germany (vfa non-interventional studies registry 15960N). Data from 396 patients were analyzed.

**Results:**

At baseline, the mean GAF score was 47.7 (SD 13.4). During 6 months of treatment with AOM, the mean GAF score increased to 59.4 (SD 15.8). Subgroups stratified by patient age (≤35 years/>35 years), sex, disease duration (≤5 years/>5 years) and disease severity at baseline had all significantly improved their GAF at month 6. 51.5% of the patients showed a GAF score increase of at least 10 points, which was regarded as clinically meaningful, and were considered responders.

**Conclusions:**

These data show that treatment with AOM may help improve patient functioning in a routine treatment setting.

**Trial registration:**

NCT02131415 (May 6, 2014), vfa non-interventional studies registry 15960N.

## Background

Functioning is impaired in many patients with schizophrenia. Even so that impairment of functioning in schizophrenia is difficult to treat, it should be a priority target for interventions [1], as this is an area of great unmet need [2]. A meta-analysis that investigated how many patients show adequate psychosocial functioning together with symptomatic remission for a timespan of at least 2 years found that this was only the case for 13.5% of patients [3].

Aripiprazole once-monthly (AOM) is an atypical antipsychotic in a long-acting injectable formulation, which provides reliable medication delivery, stable pharmacokinetics, and a means of monitoring for non-adherence [4]. The efficacy of AOM for relapse prevention in schizophrenia was demonstrated in two pivotal randomized controlled trials (RCTs) over 52 and 38 weeks respectively [5, 6]. In these trials, patient functioning was assessed using the Personal and Social Performance (PSP) scale [7] in patients previously stabilized with oral aripiprazole. In the 52-week study, mean total PSP scores from baseline were significantly worsened from baseline with placebo compared to AOM in the double-blind treatment phase [8]. In the 38-week study, mean PSP total scores worsened significantly from baseline with AOM 50 mg (subtherapeutic dose) compared to AOM 400 mg [8]. However, RCTs provide a somewhat artificial treatment setting and exclude the wide range of patients that are treated in real life, such as those with comorbidities such as depression and anxiety who require multiple medications to treat their comorbidities, or patients treated with multiple antipsychotics [9]. A recent cohort study has found that 79% of patients with schizophrenia encountered in real life would be ineligible for RCTs [10]. This renders the generalizability of the findings questionable in clinical practice. Therefore, real-world studies are an important complement to RCTs. In several real-world studies, patients with schizophrenia were found to show improvements in functioning over time when treated with AOM [11].

A review of functional outcomes in real-world studies with AOM has recently been published [11]. The author reviewed 8 articles and concludes that several studies have found improvements with AOM treatment using various measures. We aimed to expand these findings by pooling and re-analyzing data on Global Assessment of Functioning (GAF).

Here, we present pooled data from two observational studies conducted in Germany [12, 13] and Canada [14] that assessed the functioning of outpatients with schizophrenia treated with AOM using the Global Assessment of Functioning (GAF) scale. Analyses of effectiveness outcomes have already been reported: for the total population, the mean Brief Psychiatric Rating Scale total score decreased , indicating that patients on average were moderately to markedly ill at baseline, and improved to mild to moderate illness severity at month 6. This was supported by corresponding Clinical Global Impression – Severity values [15]. The main objective of the current paper was to determine whether the GAF scores improved over this 6-month period. The secondary-exploratory objectives were to examine whether the changes in GAF scores occurred in subgroups of patients defined according to age, male or female patients, duration of illness and severity of illness, which was made possible by the increased sample size resulting from analysis of both studies together. Additionally, we focused on response and remission of patient functioning, which are patient-relevant outcomes that had not been addressed previously.

## Methods

We set out to make pooled analyses of GAF data from prospective real-world observational studies in which patients with schizophrenia were treated with AOM.

We conducted *post-hoc* analyses of pooled data from two prospective observational studies in which patients with schizophrenia from Canada (NCT02131415) [14] and Germany (vfa non-interventional studies registry 15960N) [12, 13] were treated with AOM.

Both of these studies used similar designs, were observational, were performed under similar conditions, and used the same rating scales. Before the results were pooled, we undertook a feasibility analysis that analyzed the baseline data and outcomes of both studies to assess if there were any cofounding factors which may influence the pooled results. Descriptive statistical analysis and clinical discussions revealed that pooling the data would produce valid and clinically meaningful results.

We found no other studies for pooling, since no other study used a sufficiently similar study design and had the same endpoints.

In the current analysis, we included all patients who had received AOM treatment and for whom a GAF assessment at baseline and at least one time point post-baseline was available. 396 patients (228 out of 242 from Germany, 168 out of 169 from Canada) were therefore included in the analysis.

In the Canadian observational study, adult patients (at least 19 years old for patients from British Columbia) who were at least mildly ill (Clinical Global Impression – Severity [CGI-S]-score of at least 3) could be included after the treating physician had prescribed AOM. The patients were treated at 17 Canadian community or hospital-based centers. Patients who were unable to provide informed consent, presented contraindications for AOM, had been treated with AOM previously, showed significant suicidal risk, or were pregnant or lactating, were excluded from the study. AOM treatment was to be initiated as per Canadian product label. For patients with no prior use of aripiprazole, tolerability had to be established with oral aripiprazole. After the first injection, oral aripiprazole was to be taken concomitantly for 2 weeks and then discontinued. The study was originally designed to assess functioning outcome at the end of 2 years. However, it was terminated early after at least 50% of the initially planned number of patients had completed the 12-month assessment. This decision was made by the study sponsor in consultation with the investigators. In the present analyses, only data from the first 6 months were extracted to make pooling with the German study data feasible.

In the German observational study, outpatients who had been diagnosed with schizophrenia according to ICD-10 were eligible for inclusion if they were currently treated with a fixed dose of oral aripiprazole as per German product label. Patients who presented with contraindications for AOM, were members or were related to a member of the study staff, were pregnant or planned a pregnancy, were breastfeeding, or were expected to show reluctance to follow the prespecified monitoring plan (as assessed by the treating psychiatrist), were excluded from the study. The treating physician decided on the switch to AOM. Patients were to be switched to AOM as per German product label, i.e. patients had to be treated with a fixed dose of oral aripiprazole before the first injection and be considered clinically stable. After the first AOM injection, oral aripiprazole was to be taken concomitantly for 2 weeks and then discontinued. 75 treatment centers in Germany provided participant data. The planned study duration was 6 months.

GAF was an endpoint in both studies. GAF data from visits at baseline, month 3 and month 6 were pooled from both studies and are analyzed here. The GAF rates the individual’s overall functioning in the form of a single value between 1 and 100 (a score of 0 indicates insufficient information). The scale range is grouped in intervals of 10 points each. Descriptors are given in text form for each interval, with the lowest (1-10 points) described as “patient is in persistent danger of hurting self or others, or is persistently unable to maintain minimal personal hygiene, or recently performed a serious suicidal act with a clear expectation of death” and the highest (91-100 points) described as “superior functioning in a wide range of activities, no symptoms”. The treating physician is asked to first identify the appropriate 10-point interval and then select a score within the interval.

We defined a response on the GAF as an improvement by at least 10 points, matching the descriptor intervals on the GAF scale. In additional sensitivity analyses, we also looked at patients who reached a new 10-point interval on the GAF, irrespective of the number of points gained. For instance, a patient starting at 48 points at baseline and reaching 52 points at month 6 would be counted in this sensitivity analysis. Possible criteria for functional remission on the GAF may be scores of >60 [16, 17], or >80 points [18, 19]. We therefore analyzed the proportion of patients reaching these scores at 6 months.

We stratified by age using a cut-off at 35 years, because previous data have indicated that that patients up to 35 years of age may experience special benefits from AOM treatment, as shown for quality of life outcomes [20].

We used the Wilcoxon signed rank test for paired samples, and the Wilcoxon rank sum test for independent samples. Missing values were imputed using the Last Observation Carried Forward (LOCF) method if there was a value for baseline and at least one post-baseline time point. In this case, the last recorded value was used for all following time points. All tests were two-sided with alpha = 0.05, with no correction for multiple testing for secondary outcomes. Subgroups were defined according to age (according to the ≤35 and >35-year cutoff used in the QUAlity of LIFe with AbiliFY Maintena® (QUALIFY) study [20]), sex, disease duration (≤5 years and >5 years) and disease severity defined according to the baseline CGI ratings.

## Results

GAF scores were available for 396 patients. Demographics and baseline characteristics for these patients are shown in Table 1.

**Table 1:** Demographics and baseline characteristics of patients with evaluable GAF scores

	Total population (*n* = 396)
Age (years), mean (SD)	38.7 (14.6)
Sex male, n (%)	238 (60.1)
Body Mass Index (kg/m^2^), mean (SD)	29.2 (6.9)
Age at diagnosis (years), mean (SD)	29.1 (11.6)
Duration of disease since diagnosis (years), mean (SD)	9.6 (10.2)
BPRS score at baseline, mean (SD)	48.1 (15.6)
CGI-S score at baseline, mean (SD)	4.47 (0.90)
GAF score at baseline, mean (SD)	47.7 (13.4)

*BPRS* Brief Psychiatric Rating Scale, *CGI-S* Clinical Global Impression – Severity, *GAF* Global Assessment of Functioning, *SD* Standard deviation

81.8% of the analyzed population received substances for treatment of their schizophrenia in addition to AOM at study start. For most patients, this was oral aripiprazole (*n* = 305, 77.0%). Other substances that more than 2% of patients received are given in Table 2. Patient received up to 5 concomitant medications.

**Table 2:** Additional substances to treat schizophrenia at study start

Substance	Patients, n (%) (*n* = 396)
Quetiapine	25 (6.3)
Olanzapine	21 (5.3)
Clozapine	12 (3.0)
Risperidone	12 (3.0)

Comorbidities were present in 251 patients (63.4%). Up to 13 comorbidities were noted per patient. The most frequent comorbidities that were present in more than 5% of the patients are given in Table 3.

**Table 3:** Comorbidities present in more than 5% of patients at study start

Comorbidities	Patients, n (%) (*n* = 396)
Depression	53 (13.4)
Hypertension	34 (8.6)
Obesity	29 (7.3)
Anxiety	28 (7.1)
Somnolence	27 (6.8)
Blood prolactin increased	21 (5.3)
Extrapyramidal disorder	21 (5.3)
Weight increased	20 (5.1)

We compared the data of the original studies to see if they were similar enough to support pooling. Relevant GAF data are given in Table 4.

**Table 4:** GAF data from the original studies

Patients with baseline assessment	Patients from Canadian study (*n* = 169)	Patients from German study (*n* = 238)	*p*-value
GAF at baseline, mean (SD)	48.7 (12.6)	46.9 (13.9)	
Patients with baseline and at least one post-baseline assessment	Patients from Canadian NIS (*n* = 168)	Patients from German NIS (*n* = 228)	
GAF at baseline, mean (SD)	48.7 (12.6)	47.0 (13.9)	0.22^a^
GAF at month 6, mean (SD)	58.0 (13.8)	60.5 (17.1)	0.04^a^
Responders with improvement by at least 10 points, n (%)	75 (44.6)	129 (56.6)	0.02^b^

*GAF* Global Assessment of Functioning, *SD* standard deviation

^a^Wilcoxon Two-sample test; ^b^Fisher’s exact test

### Total population

**Fig. 1:**
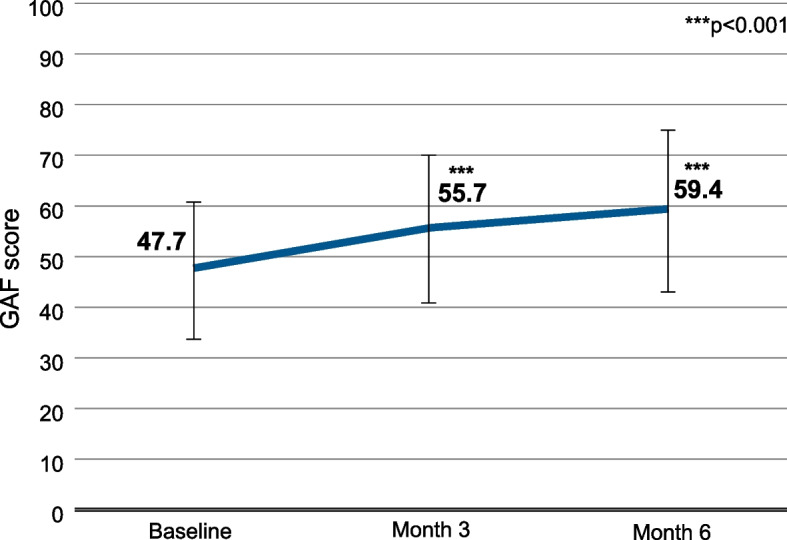
GAF scores of the total population (*n* = 396). Error bars represent standard deviations. Missing data were imputed using the Last Observation Carried Forward (LOCF) method. ***, *p* < 0.001 vs. baseline

In the analyzed population (*n* = 396), the mean GAF score at baseline was 47.7 (SD 13.4) [95% confidence interval [95% CI], 46.4-49.0] (Fig. 1). During 6 months of treatment with AOM, the mean GAF score increased to 59.4 (SD 15.8) [95% CI, 57.9-61.0]. Compared to baseline, improvements at month 3 and month 6 were statistically significant (*p* < 0.001).

### Patients ≤35 years and >35 years

**Fig. 2:**
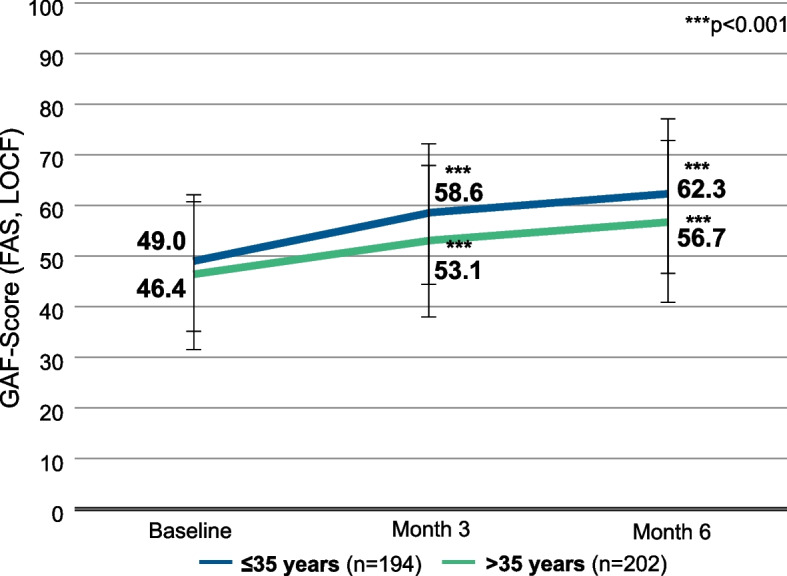
GAF scores, stratified by age. Error bars represent standard deviations. Missing data were imputed using the Last Observation Carried Forward (LOCF) method. ***, *p* < 0.001 vs. baseline

Patients ≤35 years started out with a mean GAF score of 49.0 (SD 12.7) [95% CI, 47.2-50.8] at baseline, while patients >35 years had a mean GAF score of 46.4 (SD 13.9) [95% CI, 44.5-48.3] (Fig. 2). Patients ≤35 years improved by 13.3 points (SD 16.4) [95% CI, 10.9-15.6] during 6 months of AOM treatment, reaching a mean score of 62.3 (SD 15.1) [95% CI, 60.1-64.4], compared to an improvement of 10.3 points (SD 13.9) [95% CI, 8.4-12.2] in patients >35 years, who reached a mean score of 56.7 (SD 16.1) [95% CI, 54.5-59.0].

### Patient sex

**Fig. 3:**
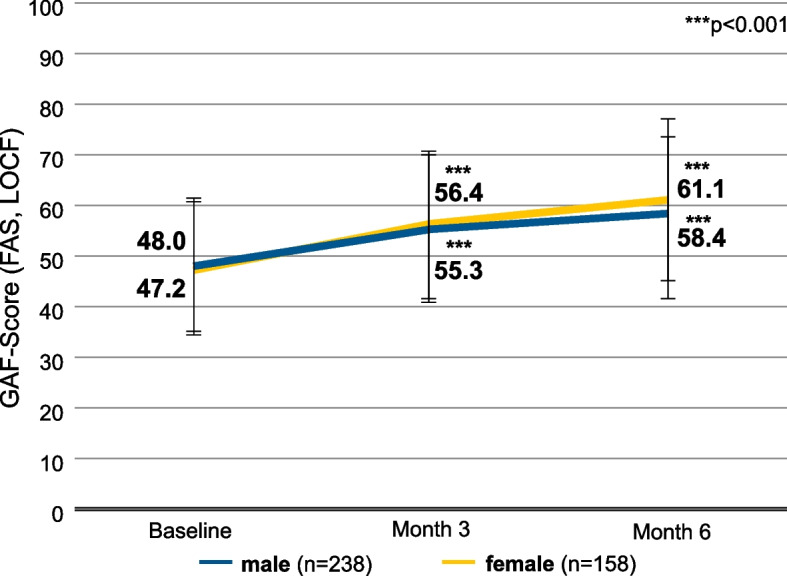
GAF scores, stratified by gendersex. Error bars represent standard deviations. Missing data were imputed using the Last Observation Carried Forward (LOCF) method. ***, *p* < 0.001 vs. baseline

At baseline, male patients had a mean GAF score of 48.0 (SD 13.4) [95% CI, 46.3-49.7], while female patients had a mean GAF score of 47.2 (SD 13.3) [95% CI, 45.1-49.3] (Fig. 3). During 6 months of treatment with AOM, male patients improved by 10.3 points (SD 14.8) [95% CI, 8.5-12.2] and reached a mean score of 58.4 (SD 15.8) [95% CI, 56.3-60.4], while female patients improved by 13.9 points (SD 15.8) [95% CI, 11.4-16.4] and reached a mean score of 61.1 (SD 15.8) [95% CI, 58.6-63.5].

### Disease duration

**Fig. 4:**
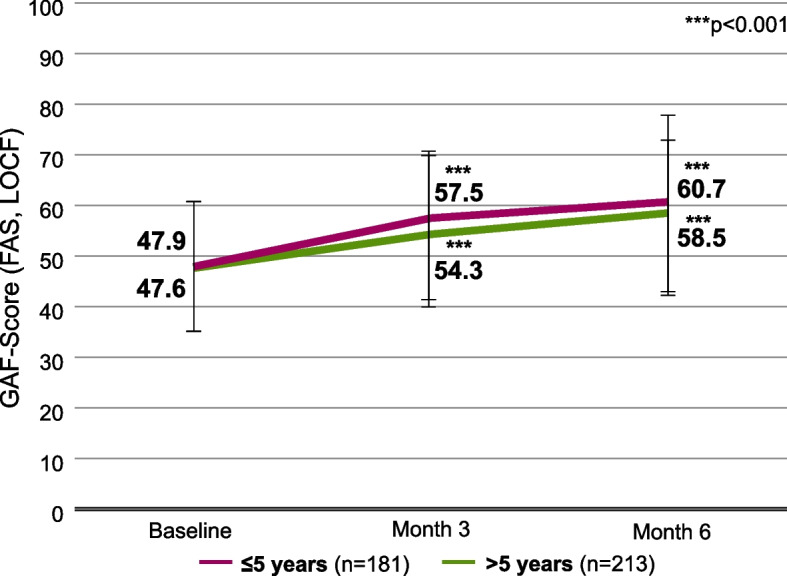
GAF scores, stratified by duration of disease. Error bars represent standard deviations. Missing data were imputed using the Last Observation Carried Forward (LOCF) method. ***, *p* < 0.001 vs. baseline

Patients with a disease duration of ≤5 years had a mean GAF score of 47.9 (SD 13.5) [95% CI, 45.9-49.9] at baseline, while patients with a disease duration of >5 years had a mean GAF score of 47.6 (SD 13.3) [95% CI, 45.8-49.4] (Fig. 4). Patients with a disease duration of ≤5 years improved by 12.8 points (SD 16.3) [95% CI, 10.4-15.2] during 6 months of AOM treatment, reaching a mean score of 60.7 (SD 15.9) [95% CI, 58.4-63.0]. Patients with a disease duration of >5 years improved by 10.9 (SD 14.3) [95% CI, 9.0-12.8], reaching a mean of 58.5 (SD 15.6) [95% CI, 56.4-60.6] at month 6.

### Baseline severity

**Fig. 5:**
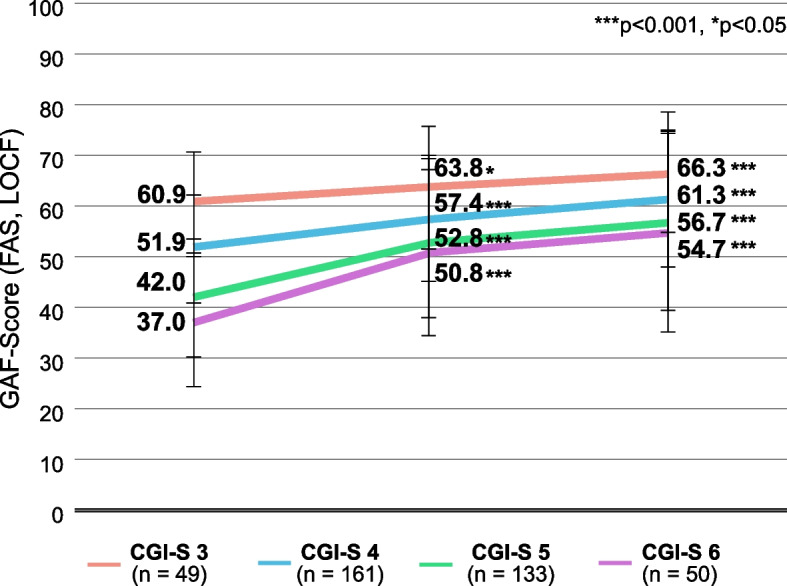
GAF scores, stratified by disease severity (CGI-S) at baseline. 1 patient with CGI-S = 1 and 2 patients with CGI-S = 7 are not shown. Error bars represent standard deviations. Missing data were imputed using the Last Observation Carried Forward (LOCF) method. ***, *p* < 0.001 vs. baseline

Patients with greater disease severity at baseline had on average lower GAF scores than patients with less severe disease (Fig. 5). Patients with all levels of disease severity improved on the GAF during treatment, and intergroup differences decreased during treatment. Whereas higher CGI scores seem to correlate with greater improvements on the GAF scale, no significance was determined.

### Responders

**Fig. 6:**
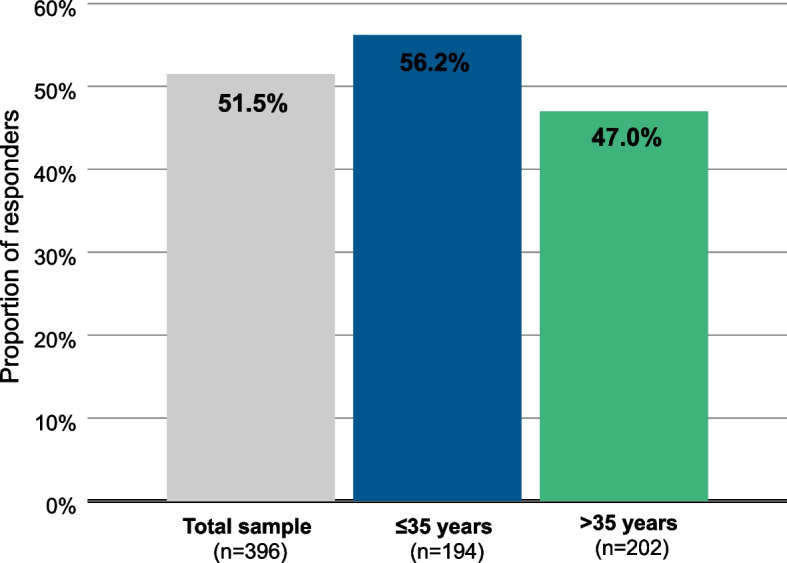
Proportion of GAF responders. Patients were considered “responders”, if they showed an improvement of at least 10 points, which corresponds to a descriptor interval on the GAF

51.5% of the patients improved by at least 10 points, which corresponds to one descriptor interval on the GAF during 6 months, and were considered “responders” (Fig. 6). Among patients ≤35 years, 56.2% achieved a 10-point improvement, and among patients >35 years this was the case for 47.0%. Sensitivity analyses based on higher cut-offs revealed that 96 patients (24.2%) achieved an improvement of 20 points or more, 60 patients (15.2%) 30 points or more, and 28 patients (7.1%) 40 points or more.

For further sensitivity analysis, we looked at the proportion of patients in the total sample that moved to a higher descriptor interval during 6 months, irrespective of the actual number of points gained. This was the case for 250 patients (63.1%). 114 patients (28.8%) remained within the same interval during 6 months, and 32 patients (8.1%) moved to a lower interval. These changes did not depend on the baseline GAF score.

### Composite responders

**Fig. 7:**
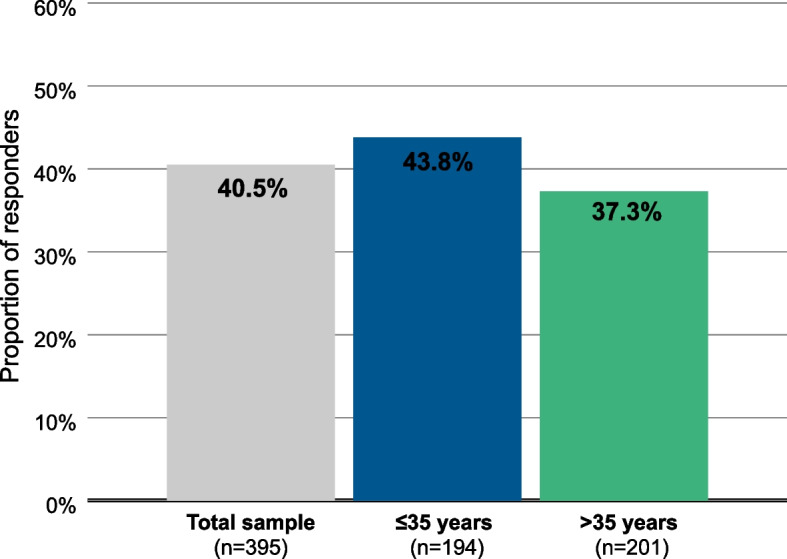
Proportion of responders on both the BPRS and GAF scales. Patients were considered “responders” if they showed an improvement of at least 20% of the BPRS total score and an improvement of at least 10 points on the GAF

40.5% of the patients were considered “responders” on both the GAF scale and the Brief Psychiatric Rating Scale (BPRS) (Fig. 7), which means that they improved by at least 10 points on the GAF and by at least 20% on the BPRS. Among patients ≤35 years of age, 43.8% met the composite response criterion, which was true of only 37.3% of patients >35 years of age.

### Functional remission

We analyzed the proportion of patients reaching >60, >70, or >80 points on the GAF at 6 months, representing possible criteria for functional remission. 179 patients (45.2%) reached >60 points, 82 patients (20.7%) reached >70 points, and 36 patients (9.1%) reached >80 points.

### Dosing

At study start, patients received a mean dose of 373.4 mg (SD, 51.0) of AOM. The majority of the patients received 400 mg (*n* = 303, 76.5%), 81 patients (20.5%) received 300 mg, and 12 (3.0%) received a lower dose. At month 6, the mean dose was 375.2 mg (SD, 73.8).

### Discontinuation

During 6 months, 44 patients (11.1%) discontinued treatment with AOM. For 11 (2.8%) of them, the reason was lack of effectiveness, 5 (1.3%) cited adverse drug reactions, and for 29 (7.3%) the declaration was “other reason”. One patient gave two reasons for discontinuation.

### Adverse events

A total of 186 patients (47.0%) of the patients experienced adverse events during the 6 month period analyzed here. Adverse events that occurred in 2% or more of the patients are given in Table 5.

**Table 5:** Adverse events that occurred in more than 1% of the patients

	Number of patients (%), *n* = 396
Any adverse event	186 (47.0)
Psychotic symptoms	28 (7.1)
Extrapyramidal symptoms	12 (3.0)
Akathisia	8 (2.0)

## Discussion

In our current analysis of pooled data from two observational studies we found that, on average, patients experienced functional improvements during 6 months of treatment with AOM. Similar results were found when subgroups of patients were analyzed. This shows that in a routine treatment setting AOM can contribute to better functional outcomes in younger as well as older patients, male as well as female patients, patients with shorter or longer disease duration and patients with various degrees of disease severity. Furthermore, many of the patients in our sample had been pre-treated with oral antipsychotics at the time of entry into the two studies, and most patients of the German population had been considered symptomatically stable at study start [12]. Under these circumstances, AOM would be expected to show a stabilizing effect that prevents patients from deteriorating. However, we saw not only maintenance of previous levels of functioning, but further improvements as well. This is a noteworthy finding.

51.5% of the patients were considered “responders” at 6 months of AOM treatment, with improvements of at least 10 points on the GAF scale. They reached a higher 10-point interval on the GAF scale, with a new descriptor, which shows that the improvement is clinically relevant and makes a difference for the patient. Response on the GAF scale was often seen together with symptomatic response (defined as ≥20% BPRS improvement). However, what constitutes a clinically relevant change on the GAF is not well defined. Amri and colleagues have proposed 4, 10 or 12 points as possible criteria for clinical relevance [21], therefore, even a difference of 4 points should be clearly discerned by the treating physician. Here, a criterion of 10 points was chosen, which seems reasonable as the GAF scale intervals span 10 points each, and it can be assumed that these are even more clearly discernible. In a sensitivity analysis, we found that 63.1% of the patients reached a higher interval on the GAF, irrespective of the number of points gained.

The definition of an adequate level of functioning is also unclear, as there are not even established standards for people not afflicted by disease [22]. Studies have used several different cutoffs on the GAF scale for functional remission, including >60 [16, 17], or >80 [18, 19] points on the GAF. In our study, 45.2% of the patients reached >60 points at month 6, the softest possible criterion, 20.7% of the patients reached >70 points, and 9.1% patients reached >80 points, fulfilling the most conservative remission criterion.

The GAF is a validated functioning scale [23] that has been shown to provide a high inter-rater and test-retest reliability [24, 25]. However, the GAF has been criticized because it may measure symptoms rather than functioning if the symptomatic burden is considered more severe than the functional impairment [24–26]. Furthermore, being a single global measure it does not capture the complexity of functioning, many areas of which may or may not be affected in patients with schizophrenia (activities of daily living, vocation, family relationships, social relationships, finances, leisure activities, self-care) [27].

### Limitations

All analyses done here have to be considered as *post-hoc*. There were some differences in baseline characteristics between the samples, with Canadian patients on average being significantly younger (*p* < 0.001) and less severely ill (*p* < 0.001) at baseline. Because of this, a feasibility analysis was done beforehand, which showed that pooling the data would produce valid results. Further limitations of our study are due to the real-world, uncontrolled, unblinded design of the original studies. A causal relationship of our results with the treatment cannot be concluded due to the lack of a comparator group. Confounders cannot be identified or excluded. Many of the patients (mainly and primarily those from Germany) had been treated with oral aripiprazole before inclusion in the study, potentially enriching the sample with patients who tolerated aripiprazole. Furthermore, patients may have had expectation bias since they were aware of the treatment and willing to try AOM. Real-world studies are nonetheless an important complement to RCTs [9, 10].

## Conclusions

Our work here showed that patients with schizophrenia, treated with AOM in everyday clinical conditions, may experience clinically relevant functional improvements. These data add support to the body of evidence demonstrating the robust effectiveness of AOM in schizophrenia. Although no significant differences between younger and older patients emerged, especially younger patients with fewer episodes and less time spent with illness probably benefit the most from the treatment with AOM.

## Data Availability

The datasets analyzed during the current study are not publicly available due to data from Canadian patients being property of Lundbeck Canada Inc. and Otsuka Canada Pharmaceutical Inc. but are available from the corresponding author on reasonable request.

## Abbreviations

AOM: Aripiprazole once-monthly
BPRS: Brief Psychiatric Rating Scale
CGI-S: Clinical Global Impression – Severity
GAF: Global Assessment of Functioning
ICD-10: International Statistical Classification of Diseases and Related Health Problems, 10th Revision
LOCF: Last Observation Carried Forward
PSP: Personal and Social Performance
QUALIFY: QUAlity of LIFe with AbiliFY Maintena®
RCT: Randomized controlled trial
REACT: REAl-world effeCTiveness of aripiprazole once-monthly
SD: Standard deviation

## References

[1] Morin L, Franck N (2017). Rehabilitation Interventions to Promote Recovery from Schizophrenia: A Systematic Review. Front Psychiatry..

[2] Giuliani L, Giordano GM, Bucci P, Pezzella P, Brando F, Galderisi S (2021). Improving knowledge on pathways to functional outcome in schizophrenia: main results from the italian network for research on psychoses. Front Psychiatry..

[3] Jääskeläinen E, Juola P, Hirvonen N, McGrath JJ, Saha S, Isohanni M (2013). A systematic review and meta-analysis of recovery in schizophrenia. Schizophr Bull..

[4] Correll CU, Citrome L, Haddad PM, Lauriello J, Olfson M, Calloway SM (2016). The Use of Long-Acting Injectable Antipsychotics in Schizophrenia: Evaluating the Evidence. J Clin Psychiatry..

[5] Kane JM, Sanchez R, Perry PP, Jin N, Johnson BR, Forbes RA (2012). Aripiprazole intramuscular depot as maintenance treatment in patients with schizophrenia: a 52-week, multicenter, randomized, double-blind, placebo-controlled study. J Clin Psychiatry..

[6] Fleischhacker WW, Sanchez R, Perry PP, Jin N, Peters-Strickland T, Johnson BR (2014). Aripiprazole once-monthly for treatment of schizophrenia: double-blind, randomised, non-inferiority study. Br J Psychiatry..

[7] Morosini PL, Magliano L, Brambilla L, Ugolini S, Pioli R (2000). Development, reliability and acceptability of a new version of the DSM-IV Social and Occupational Functioning Assessment Scale (SOFAS) to assess routine social functioning. Acta Psychiatr Scand..

[8] Fleischhacker WW, Baker RA, Eramo A, Sanchez R, Tsai L-F, Peters-Strickland T (2014). Effects of aripiprazole once-monthly on domains of personal and social performance: results from 2 multicenter, randomized, double-blind studies. Schizophr Res..

[9] Haddad PM, Tiihonen J, Haukka J, Taylor M, Patel MX, Korhonen P (2011). The place of observational studies in assessing the effectiveness of depot antipsychotics. Schizophr Res..

[10] Taipale H, Schneider-Thoma J, Pinzón-Espinosa J, Radua J, Efthimiou O, Vinkers CH (2022). Representation and outcomes of individuals with schizophrenia seen in everyday practice who are ineligible for randomized clinical trials. JAMA Psychiatry..

[11] Nick B. Aripiprazol-Depot bei Schizophrenie im Behandlungsalltag: Funktionalität und Lebensqualität. Swiss Arch Neurol Psychiatr Psychother. 2021;172.

[12] Schöttle D, Janetzky W, Luedecke D, Beck E, Correll CU, Wiedemann K (2018). Effectiveness of aripiprazole once-monthly in schizophrenia patients pretreated with oral aripiprazole: a 6-month, real-life non-interventional study. BMC Psychiatry..

[13] Schöttle D, Janetzky W, Luedecke D, Beck E, Correll CU, Wiedemann K (2020). The use of long-acting Aripiprazole in a multi-center, prospective, uncontrolled, open-label, cohort study in Germany: a report on global assessment of functioning and the WHO wellbeing index. BMC Psychiatry..

[14] Mustafa S, Bougie J, Miguelez M, Clerzius G, Rampakakis E, Proulx J (2019). Real-life assessment of aripiprazole monthly (Abilify Maintena) in schizophrenia: a Canadian naturalistic non-interventional prospective cohort study. BMC Psychiatry..

[15] Schöttle D, Clerzius G, Janetzky W, Oluboka O, Roy M-A, Therrien F (2022). Real-world effectiveness of aripiprazole once-monthly REACT study: Pooled analysis of two noninterventional studies. Eur Psychiatry..

[16] Boyer L, Richieri R, Guedj E, Faget-Agius C, Loundou A, Llorca P-M (2013). Validation of a functional remission threshold for the Functional Remission of General Schizophrenia (FROGS) scale. Compr Psychiatry..

[17] Valencia M, Fresán A, Barak Y, Juárez F, Escamilla R, Saracco R (2015). Predicting functional remission in patients with schizophrenia: a cross-sectional study of symptomatic remission, psychosocial remission, functioning, and clinical outcome. Neuropsychiatr Dis Treat..

[18] Bobes J, Ciudad A, Alvarez E, San L, Polavieja P, Gilaberte I (2009). Recovery from schizophrenia: results from a 1-year follow-up observational study of patients in symptomatic remission. Schizophr Res..

[19] San L, Ciudad A, Alvarez E, Bobes J, Gilaberte I (2007). Symptomatic remission and social/vocational functioning in outpatients with schizophrenia: prevalence and associations in a cross-sectional study. Eur Psychiatry..

[20] Naber D, Hansen K, Forray C, Baker RA, Sapin C, Beillat M (2015). Qualify: a randomized head-to-head study of aripiprazole once-monthly and paliperidone palmitate in the treatment of schizophrenia. Schizophr Res..

[21] Amri I, Millier A, Toumi M (2014). Minimum Clinically Important Difference in the Global Assessment Functioning in Patients with Schizophrenia. Value Health..

[22] Harvey PD, Bellack AS (2009). Toward a terminology for functional recovery in schizophrenia: is functional remission a viable concept?. Schizophr Bull..

[23] Jones SH, Thornicroft G, Coffey M, Dunn G (1995). A brief mental health outcome scale-reliability and validity of the Global Assessment of Functioning (GAF). Br J Psychiatry..

[24] Smith GN, Ehmann TS, Flynn SW, MacEwan GW, Tee K, Kopala LC (2011). The assessment of symptom severity and functional impairment with DSM-IV axis V. Psychiatr Serv..

[25] Gaite L, Vázquez-Barquero JL, Herrán A, Thornicroft G, Becker T, Sierra-Biddle D (2005). Main determinants of Global Assessment of Functioning score in schizophrenia: a European multicenter study. Compr Psychiatry..

[26] Suzuki T, Uchida H, Sakurai H, Ishizuki T, Tsunoda K, Takeuchi H (2015). Relationships between global assessment of functioning and other rating scales in clinical trials for schizophrenia. Psychiatry Res..

[27] Brown MA, Velligan DI (2016). Issues and developments related to assessing function in serious mental illness. Dialogues Clin Neurosci..

